# A Large Spatial Survey of Colistin-Resistant Gene *mcr-1*-Carrying *E. coli* in Rivers across Taiwan

**DOI:** 10.3390/microorganisms9040722

**Published:** 2021-03-31

**Authors:** Ching-Hao Teng, Pin-Chieh Wu, Sen-Lin Tang, Yi-Chen Chen, Ming-Fang Cheng, Ping-Chih Huang, Wen-Chien Ko, Jiun-Ling Wang

**Affiliations:** 1 Institute of Molecular Medicine, College of Medicine, National Cheng Kung University, Tainan 704, Taiwan; chteng@ncku.edu.tw (C.-H.T.); cfdsa85213@gmail.com (Y.-C.C.); 2 Institute of Basic Medical Sciences, College of Medicine, National Cheng Kung University, Tainan 704, Taiwan; 3 Center of Infectious Disease and Signaling Research, National Cheng Kung University, Tainan 704, Taiwan; 4 Department of Physical Examination Center, Kaohsiung Veterans General Hospital, Kaohsiung 813, Taiwan; wpchieh@vghks.gov.tw; 5 Department of Nursing, Meiho University, Pingtung 912, Taiwan; 6 Biodiversity Research Center, Academia Sinica, Taipei 115, Taiwan; sltang@gate.sinica.edu.tw; 7 Department of Pediatrics, Kaohsiung Veterans General Hospital, Kaohsiung 813, Taiwan; mfcheng@vghks.gov.tw; 8 School of Medicine, National Yang-Ming University, Taipei 112, Taiwan; 9 Department of Chemical Engineering, Institute of Biotechnology and Chemical Engineering, I-Shou University, Kaohsiung 840, Taiwan; 10 Department of Nursing, Fooyin University, Kaohsiung 831, Taiwan; 11 Department of Cosmetics and Fashion Styling, Cheng-Shiu University, Kaohsiung 833, Taiwan; pch@mail.csu.edu.tw; 12 Department of Internal Medicine, National Cheng Kung University Hospital, Tainan 704, Taiwan; winston3415@gmail.com; 13 Department of Medicine, College of Medicine, National Cheng Kung University, Tainan 704, Taiwan

**Keywords:** river, plasmid, *mcr-1*, chicken, pigs, transconjugants, livestock density

## Abstract

Background: Colistin is one of the last-line antimicrobial agents against life-threatening infections. The distribution of the colistin resistance gene *mcr-1* has been reported worldwide. However, most studies have focused on the distribution of *mcr-1*-positive bacteria in humans, animals, food, and sewage; few have focused on their distribution in natural environments. Method: We conducted a large spatial survey of *mcr-1*-positive *Escherichia coli* at 119 sites in 48 rivers, covering the entire island of Taiwan. We investigated the relationship between the livestock or poultry density in the surveyed riverine area and the number of *mcr-1*-positive *E. coli* in the river water. We then sequenced and characterized the isolated *mcr-1*-positive plasmids. Results: Seven *mcr-1* positive *E. coli* were isolated from 5.9% of the sampling sites. The *mcr-1*-positive sites correlated with high chicken and pig stocking densities but not human population density or other river parameters. Four of the *mcr-1*-positive *E. coli* strains harbored epidemic IncX4 plasmids, and three of them exhibited identical sequences with a size of 33,309 bp. One of the plasmids contained identical 33,309 bp sequences but carried an additional 5711-bp transposon (Tn3 family). To our knowledge, this is the first demonstration that *mcr-1*-carrying IncX4 plasmids can contain an insertion of such transposons. All *mcr-1*-positive isolates belonged to phylogenetic group A and harbored few known virulence genes. Conclusion: This study showed a positive relationship between the number of *mcr-1*-positive sites and high livestock and poultry density. The sequencing analyses indicated that the epidemic plasmid in the *mcr-1* isolates circulates not only in humans, animals, and food but also in the associated environments or natural habitats in Taiwan, suggesting that the surveillance of antibiotics-resistance genes for livestock or poultry farm quality control should include their associated environments.

## 1. Introduction

The rapid emergence and dissemination of antibiotic-resistant bacteria has become a serious threat to public health globally. Colistin is one of the last-line antimicrobial agents for treating life-threatening infections caused by multidrug-resistant bacteria. However, after the first report by Lui in China in 2015, the plasmid-mediated colistin resistance gene *mcr-1* has been found to be widespread in five continents [1,2]. The marked increase in *mcr-1*-carrying bacterial isolates worldwide can be explained by the initial mobilization of *mcr-1* by an ISApl1 transposon in the mid-2000s and its rapid mobilization onto other plasmids [3]. *Escherichia coli* is the most prevalent species among the *mcr-1*-positive isolates, and constitutes approximately 90% of the total *mcr-1*-carrying isolates [2]. Additionally, *mcr-1* carrying isolates can be found in raw meat, livestock animals, infections, and healthy people [1,4]. A higher number or percentage of *mcr-1* carriage in *E. coli* isolates has been found in raw meat and food animals than in human isolates [1]. Researchers suggest that *mcr-1* resistance may have emerged in the animal sector due to the widespread use of colistin in food animals, including pig and poultry farms, in several areas [5]. Additionally, a study conducted in China found that 6% of human fecal samples carried the *mcr-1* gene [6]. Colistin is prohibited as a growth promoter in Taiwan, but it can be used for disease treatment in veterinary treatment [7]. In Taiwan, *mcr-1*-positive *E. coli* has been found in 0.4% of asymptomatic adults [8] and 0.6% of clinical isolates [9]. Similar to studies in other countries, the rate of *mcr-1* positive isolates from meat and swine/poultry diseases in Taiwan exceeds that in isolates from humans (retail meats: 8.7% in 2015; diseased swine: 33.3% in 2016) [7,10].

In addition to in food animals and humans, *mcr-1* has been found in aquatic systems, particularly sewage or wastewater [11,12]. A recent literature review indicated that 18 publications have reported on plasmid-mediated colistin resistance in 2107 isolates from freshwater and seawater [13], though few have focused on *mcr-1*-positive isolates in natural habitats or associated environments [13]. In addition to China [5,14,15], *mcr-1* has also been observed in freshwater environments in Malaysia [16], Italy [17], and Switzerland [18]. However, these studies have mainly focused on one or a few freshwater sites.

To gain a full picture of the distribution of *mcr-1*-positive *E. coli* in Taiwan’s freshwater system, an intensive survey in 52 rivers across the entire island of Taiwan was conducted in this study. Taiwan is a mountainous island, consisting of 268 mountains above 3000 m, with most of the rivers flowing in a steep descent from the center of the island to the surrounding sea [19]. In addition to comprehensively surveying *mcr-1*-positive *E. coli*, this survey allowed us to examine our hypothesis that the number of *mcr-1* isolates in the river could be correlated with the livestock or poultry density in the riverine environment. Moreover, the plasmid sequences and their gene content in the positive *E. coli* provided more insight into the ecology of colistin-resistant genes in Taiwan and the Western Pacific region.

## 2. Materials and Methods

### 2.1. Water Sampling

**Figure 1:**
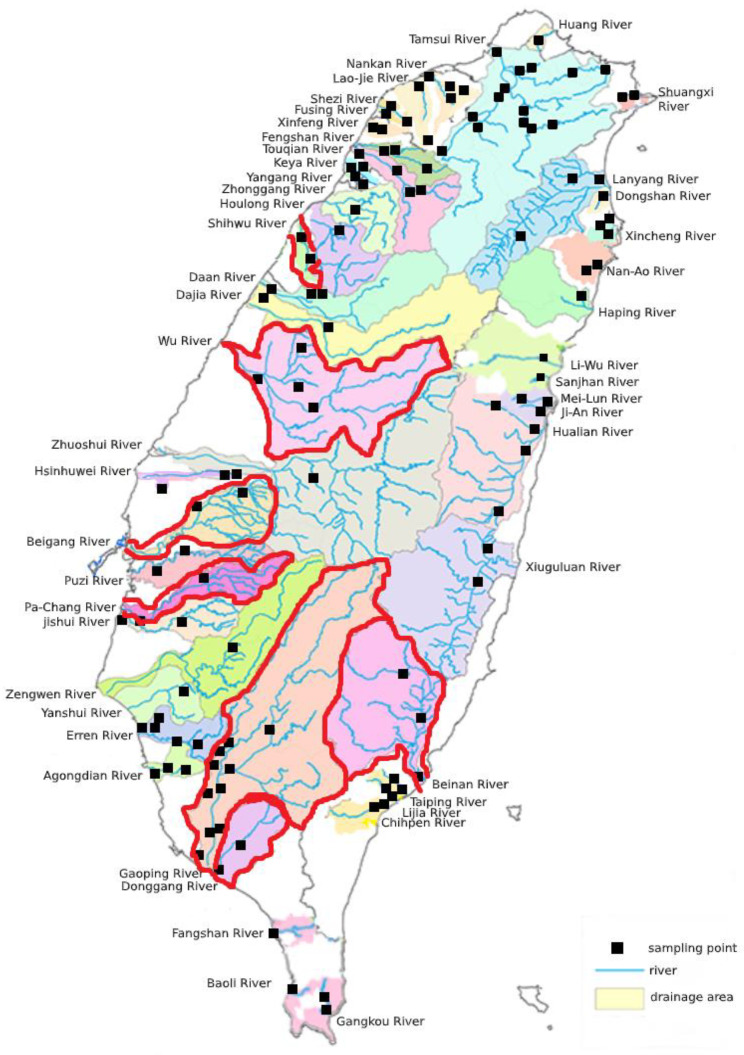
River collection sites in this study; riverine areas with *mcr-1*-positive sites are indicated by a red line.

Water samples were collected from different rivers from December 2015 to February 2016 (Figure 1). The Taiwan Environmental Protection Administration (TEPA) routinely examines river water in Taiwan to monitor the river pollution index and coliform bacteria population [20,21]. The river pollution index includes the concentrations of four parameters in water: dissolved oxygen (DO), biochemical oxygen demand (BOD5), suspended solids (SS), and ammonia nitrogen (NH_3_-N). Information regarding the livestock and poultry stocking numbers was obtained from the Council of Agriculture, Taiwan ((https://agrstat.coa.gov.tw/sdweb/public/book/Book.aspx) (accessed on 30 March 2021) and (https://www.naif.org.tw/main.aspx) (accessed on 30 March 2021). A total of 48 rivers and 119 sites were sampled in this study. We sampled each river site three times during the study period. Up to two isolates of *E. coli* were cultured from each sampling site. Therefore, a total of 537 *E. coli* isolates were obtained.

One-hundred milliliters of river water was sampled each time and analyzed following the standard procedures of the Environmental Analysis Laboratory of the TEPA [22]. Additionally, all of the sampling procedures followed the rules established by the Environmental Analysis Laboratory of the TEPA. TEPA method: River, Lake, Reservoir water quality sampling general rule, National Institute of Environmental Analysis (NIEA)W104.51C was used as the standard basis for sampling. The water samples were analyzed immediately after collection, and the time between sample collection to laboratory work completion was <24 h.

To conduct cluster sampling for each river and county, we randomly selected *E. coli* from samples collected at the 119 river stations in Taiwan. One water sample was collected and filtered to further isolate *E. coli* at each sampled station, following previously described methods [22]. We used *E. coli* CHROMagar (ECC) plates (CHROMagar, Paris, France) to screen *E. coli*, which was incubated at 37 °C for 24 h, and up to two *E. coli* colonies were selected per sample. *mcr-1* PCR was conducted in *E. coli* isolates using the primers CLR5-F (5′-CGGTCAGTCCGTTTGTTC-3ʹ) and CLR5-R (5′-CTTGGTCGGTCTGTA GGG-3′), following previously described methods [1]. We used eight housekeeping gene sequences (*adk*, *fumC*, *gyrB*, *icd, mdh*, *purA*, and *recA*) according to the protocol on the MLST database website (http://mlst.warwick.ac.uk/mlst/dbs/Ecoli) (accessed on 30 March 2021), and the broth dilution method, to check the antimicrobial susceptibility of sulfamethoxazole and trimethoprim (STX-TMZ), ciprofloxacin, tetracycline, meropenem, azithromycin, nalidixic acid, cefotaxime, chloramphenicol, tigecycline, ceftazidime, colistin, ampicillin, and gentamicin (Clinical and Laboratory Standards Institute (CLSI), 2020). Colistin was defined as intermediate (MICs (minimum inhibitory concentrations) of ≤2 mg/L) or resistant (MICs ≥ 4 mg/L) according to the MIC interpretive criteria from the CLSI.

### 2.2. Conjugation Assays

Conjugation assays were conducted to determine whether the *mcr-1* genes in the river isolates were harbored on conjugative plasmids. The *E. coli* strains MG1655 *lacZ::*Gm and MG1655 *recA::*Tet, which harbor gentamycin and tetracycline resistance, respectively, were used as recipients in the assays. The five colistin-resistant river isolates (EC1278, EC1279, EC1280, EC1281, and EC1283) served as donors. Based on the antibiotic resistance, MG1655 *lacZ::*Gm was used as the recipient in the conjugation experiment with EC1278, EC1279, EC1281, and EC1283, while MG1655 *recA::*Tet was used as the recipient of EC1280. The bacterial strains were grown overnight in Lysogeny broth (LB) medium containing appropriate antibiotics (2 mg/mL of colistin, 2 mg/mL of gentamycin, or 2 mg/mL of tetracycline) [23]. To remove antibiotics from the overnight cultures, the bacteria in 200 μL samples of the cultures were washed with 200 μL of fresh LB medium once by centrifugation at 1000× *g* for 3 min and then re-suspended in 20 μL of fresh LB medium. The resulting donor (20 μL) and recipient (20 μL) suspensions were mixed, and 10 μL of the mixture was dropped on an LB agar plate without any antibiotic. After incubation at 37 °C for 24 h, the bacteria on the plate were re-suspended in PBS and spread on LB plates containing colistin, gentamycin, or tetracycline, to select for colistin-resistant transconjugants. Colistin had the growth inhibition of the recipients, while gentamycin or tetracycline had the growth inhibition of the donors.

### 2.3. Purification, Sequencing, and Assembly of Plasmids

To determine plasmid patterns in bacteria, plasmids were isolated following the alkaline method of Kado and Liu [24] and subjected to agarose gel electrophoresis.

To sequence the colistin-resistant plasmids, the plasmids were purified from the responding transconjugants following the alkaline lysis method described previously [25]. The complete nucleotide sequences of the plasmids were determined by MiSeq sequencing (Illumina Inc., San Diego, CA, USA.). The plasmids were annotated using the DDBJ (DNA Data Bank of Japan) Fast Annotation and Submission Tool (DFAST) pipeline [26]. Insertion sequences (IS) were annotated using ISFinder (https://isfinder.biotoul.fr/) (accessed on 30 March 2021), as described previously [27]. For comparative analysis, plasmid sequences were aligned against the non-redundant database using the MegaBLAST algorithm (NCBI BLAST), with the default settings for the parameters.

### 2.4. PCR-Based Phylogenetic Typing and Genotyping

The phylogenetic types of the river colistin-resistant *E. coli* strains were determined using a triplex PCR-based method to detect the presence of the *chuA* and *yiaA* genes, and the DNA fragment TSPE4.C2, as described previously [28]. The presence of known virulence genes in *E. coli* was determined by PCR using previously described primers and conditions [29,30,31,32]. The RS218 and CFT073 pathogenic *E. coli* strains served as positive controls in the PCR analyses of *ompT*, *ibeA*, *cnf1*, *sfaS*, *ireA*, *chuA*, *ihA*, *usp*, *sat*, *iroN*, and *hlyA*. The clinical *E. coli* isolates A865, which had been previously identified as harboring *afa/draBC* [32,33,34], and EC586, which harbors *hlyF*, *iutA*, and *iss* (unpublished data), served as positive controls for the corresponding genes. Additionally, the MG1655 *E. coli* strain served as a negative control for all the genes, excluding *ompT*. An *ompT*-deletion strain of RS218 served as the negative control for *ompT*. The primer sequences used for the PCR analyses are listed in Supplementary Materials Table S1.

### 2.5. Accession Numbers

The sequences of EC1279, EC1280, EC1281, and EC1283 were deposited in GenBank under the accession numbers MW010025, MW010026, MW010024, and MW010027, respectively.

### 2.6. Statistical Analysis

All statistical analyses were conducted using SPSS version 20.0 for Windows (SPSS Inc., Armonk, NY, USA). Categorical variables were analyzed using the Chi-square or Fisher’s exact tests, and the continuous variables were analyzed by conducting an independent samples *t*-test. A *p*-value of <0.05 was considered statistically significant.

## 3. Results

### 3.1. Positive mcr-1 E. coli Sites

In the river environment, *mcr-1*-positive *E. coli* were found at 5.9% (7/119) of the water sampling sites in the rivers of Taiwan. The seven *mcr-1*-positive sites were distributed in central (*n* = 3, Wu, Shihwu, and Beigang Rivers), southern (*n* = 3, Ba-Chang, Gaoping, and Donggang Rivers), and eastern Taiwan (*n* = 1, Beinan River; Figure 1). No *mcr-1*-positive sites were identified in northern Taiwan in our survey. Excluding two sites (Wu and Beigang Rivers), most (71%; 5/7) *mcr-1* positive isolates were collected from the downstream area of the river.

An example of an *mcr-1*-positive site in the downstream area (Gaoping River) is provided in Supplementary Figure S1. For the seven isolates, the antimicrobial drug susceptibility is shown in Table 1. Two of the seven isolates were susceptible to colistin in the broth dilution method. The MLST study identified different STs, including ST155, ST6732, ST877, ST7149, and ST3661 (*n* = 3).

The site characteristics regarding the distribution of *mcr-1*-positive and negative *E. coli* are shown in Table 2. The air and water temperature, river pollution index, PH, and other parameters, such as the number of coliforms in the *mcr-1*-positive and negative groups, are shown in Table 2. The pollution index was slightly higher at the *mcr-1*-positive sites, but this difference was not statistically significant in the *t*-test. The human population density, air/water temperature, pH, and coliform number were similar between the *mcr-1*-positive and negative groups. Using a continuous variable, the livestock stocking density was higher at the *mcr-1*-positive sites but did not reach statistical significance in the independent *t*-test.

**Figure 2:**
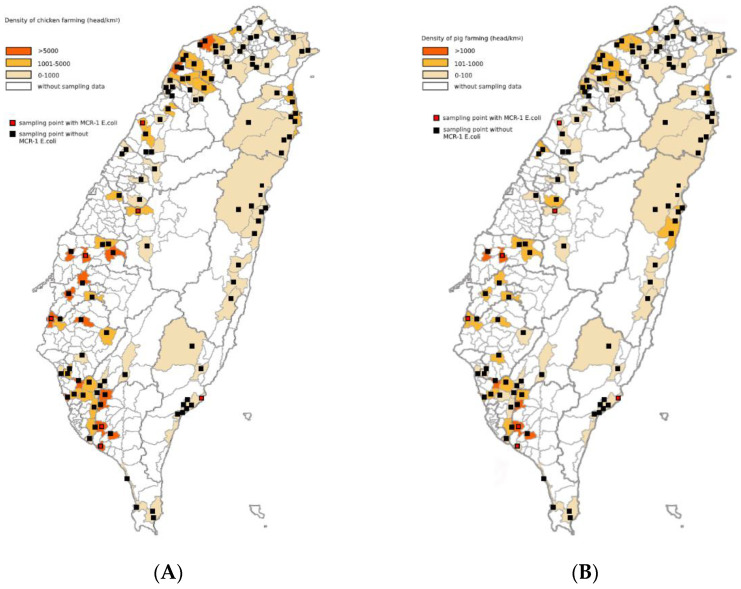
Livestock density and *mcr-1* positive and negative site correlations: (**A**) (left): chicken density; (**B**) (right) pig density.

The relationship between the *mcr-1* positive sites and chicken and pig stocking densities is shown in Figure 2A (left) and Figure 2B (right).

According to the Chi-square test using categorical variables for analysis (Table 3), *mcr-1*-positive sites were more likely to occur in the category with chicken stocking densities of 1000–5000 and >5000 birds/km^2^ (42.9% vs. 27.7%; 42.9% vs. 12.5%; *p* = 0.028) than *mcr-1*-negative sites. Regarding pig density, *mcr-1*-positive sites were more likely to occur in the category with a stocking density of >1000 herds/km^2^ (28.6% vs. 3.6%; *p* = 0.012) than *mcr-1*-negative sites (Table 3). The distributions of *mcr-1* positive and negative sites did not differ between the four pollution indices (unpolluted, negligible, moderately, and severely polluted) and human density (more or less than 1000 people/km^2^) categories (Table 3).

### 3.2. Capturing mcr-1-Carrying Conjugative Plasmids

**Figure 3:**
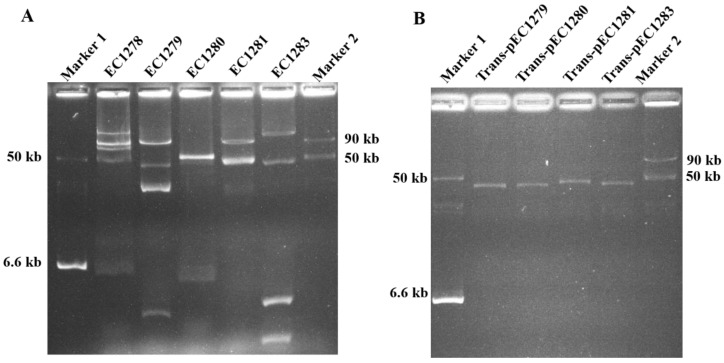
Plasmids in the colistin-resistant strains. (**A**) Plasmids in the riverine colistin-resistant *E. coli* isolates. (**B**) Plasmids in the transconjugants that obtained colistin-resistant plasmids from the riverine strains. Size markers 1 and 2 were plasmids isolated from *Salmonella* strains OU7058 and OUT7526, respectively [35].

*mcr-1* genes are often carried by conjugative plasmids. Therefore, plasmid patterns of the river colistin-resistant strains were investigated. As shown in Figure 3A, all five strains harbored multiple plasmids, and their plasmid patterns differed. The distinct plasmid patterns may reflect the distinct regions of the isolated strains.

To further investigate whether the *mcr-1* genes in the river colistin-resistant strains were encoded in conjugative plasmids, conjugation experiments were conducted with the colistin-resistant strains as donors and *E. coli* MG1655-derived strains as recipients, selecting for colistin-resistant transconjugants. The colistin-resistant transconjugants were obtained from experiments with four river-born strains, including EC1279, EC1280, EC1281, and EC1283, which were denoted as Trans-1279, Trans-1280, Trans-1281, and Trans-1283, respectively, and the plasmid profiles of the transconjugants were investigated. As shown in Figure 3B, Trans-1279, Trans-1280, and Trans-1283 harbored plasmids with similar sizes, while Trans-1281 harbored a plasmid that was apparently larger than those in the other transconjugants. These results suggest that the *mcr-1* genes in four of the river-borne colistin-resistant *E. coli* strains were encoded in conjugative plasmids.

### 3.3. Genetic Characterization of mcr-1-Carrying Plasmids

**Figure 4:**
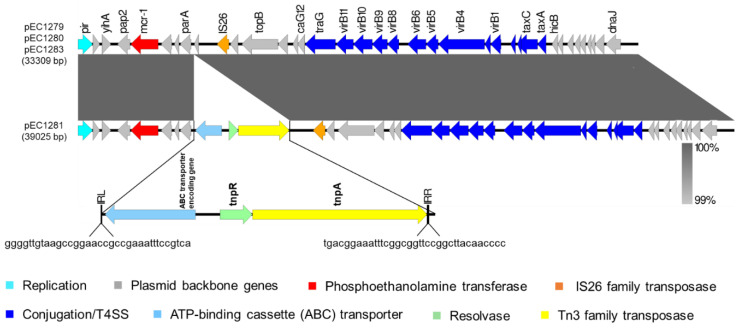
Linear comparison of the pEC1279-like plasmids (pEC1279, pEC1280, and pEC1283) and pEC1281, which contained a Tn3 family transposon. IRL—left-inverted repeated sequence; IRR—right-inverted repeated.

The colistin resistance-encoding plasmids were purified from the transconjugants and sequenced for further characterization. The plasmids derived from EC1279, EC1280, EC1281, and EC1283 were designated pEC1279, pEC1280, pEC1281, and pEC1283, respectively. pEC1279, pEC1280, and pEC1283 were 100% identical in sequence and size (33,309 bp). pEC1281 was 39,025 bp in size, which contained a 33,309 bp region identical to those of the above plasmids with the insertion of an additional 5716-bp fragment (Figure 4).

These plasmids are the IncX4 plasmids. Based on BLAST analysis, pEC1279, pEC1280, and pEC1283 aligned well with a group of IncX4 plasmids (>99.9% identity), which were characterized by harboring an *mcr-1* gene with a downstream *pap2* gene and insertion sequence IS26 located upstream of the *mcr-1*-*pap2* element (Figure 4). The plasmids in this group were mainly carried by *Enterobacteriaceae* isolated from humans, animals, meat, and wastewater, and are distributed worldwide (Table 4). We designated these plasmids as being pEC1279-like. Notably, on the island of Taiwan, pEC1279-like plasmids were identified from bacteria isolated from humans and swine, such as pNG14043 (*Salmonella* from a human), pKP15450-MCR-1 (*Klebsiella pneumoniae* from a human), and pNCYU-24-74-6_MCR1 (*E. coli* from a swine; Table 4). This study demonstrates that such *mcr-1*-carrying plasmids have spread to the natural environment of the island, in addition to humans, animals, and foods.

The additional 5.7-kb fragment in pEC1281 was located between the *mcr-1*-*pap2* element and IS26, which contained a transposon structure that encoded a transposase, resolvase, and potential ABC transporter with a 35-bp inverted repeat (IR) sequence at both ends (Figure 4). This transposon belongs to the Tn3 family [46], and has been identified in various plasmids, such as pCHL5009T-102k-*mcr*3 [46], pH226B [47], pNDM5-GZ04_A [48], and pV233-b [49]. However, to the best of our knowledge, this is the first demonstration of the insertion of a pEC1279-like *mcr-1*-carrying plasmid by this type of transposon.

### 3.4. Genetic Features of Colistin-Resistant River Isolates

To further investigate the genetic background of colistin-resistant river isolates, the phylogenetic types of EC1278, EC1297, EC1280, EC1281, and EC1283 were investigated. *E. coli* strains are primarily classified into four phylogenetic groups, designated A, B1, B2, and D [27,50]. Extraintestinal pathogenic *E. coli* (ExPEC) are mainly derived from phylogenetic groups B2 and D, while commensal *E. coli* are mainly derived from Groups A and B1 [51]. All of the strains identified here belonged to phylogenetic group A, suggesting that these river isolates may not have been pathogenic *E. coli*.

We further analyzed the virulence capability of the strains by determining the presence of 15 known *E. coli* virulence genes in the bacteria. The pathogenic roles of these virulence genes include adherence (*afa/draBC*, *iha*, and *sfaS*), invasion (*ibeA*), toxins (*cnf1, hlyA, sat,* and *hlyF*), iron uptake (*chuA*, *ireA*, *iroN,* and *iutA*), bacterial resistance to complement-mediated attack (*iss*), and miscellaneous pathogenic functions (*ompT* and *usp*). Excluding *ompT*, the known virulence genes were not detected in the five river isolates. *ompT* was identified in EC1278 and EC1283, but not in the other isolates. These findings indicate that these river isolates had low virulence, suggesting that these river colistin-resistant *E. coli* strains were non-pathogenic.

## 4. Discussion

This study demonstrated that the sites containing *mcr-1* positive *E. coli* in rivers were positively correlated with the density of livestock and poultry in the riverine area of Taiwan. No correlations were detected between pH, temperature, pollution index, and human density. Although colistin-resistant genes were only distributed in a few rivers, Taiwan’s natural environment has been contaminated with *mcr-1*-positive bacteria. Natural habitats or human activity-associated environments that have not yet been considered may serve as hidden yet critical spaces for bacterial gene transfer and transmission of resistant genes. We suggest natural environment surveys should be conducted to monitor the dissemination of colistin-resistant genes.

Contamination of *mcr-1* positive *E. coli* in rivers was likely due to anthropogenic activities, as the distribution of these bacteria was associated with high pig and poultry population densities (Figure 2). Colistin has been approved for animal use by the Council of Agriculture in Taiwan. It is likely that its use poses a selection advantage for *mcr-1*-positive *E. coli* that live in the intestines of livestock. Following population expansion, such bacteria may be released from the animals and reach and contaminate nearby rivers. Conventional livestock waste treatment processes cannot completely remove antibiotic-resistance genes, and result in contamination of water environments [52,53]. Some studies have reported the transmission of antimicrobial-resistant bacteria from pig manure to the environment [54], and these antibiotic-resistant bacteria may spread through water [55].

Our study showed that the *mcr-1*-positive sites were correlated with livestock density in river environments, and correlations between other antimicrobial resistance genes and livestock have been reported in the literature. Poultry production carries a high risk for antibiotic resistance emergence and consumes more antibiotics than the cultivation of other animals [56]. The size and scale of poultry farming are associated with the antimicrobial colonization rates [56]. Independent of antimicrobial drug usage, there is evidence of a relationship between chicken density and antimicrobial-resistant pathogens [57]. The results of the analysis of ESBL genes from chicken feces and upstream and downstream river water suggest that animal farm effluent could contribute to the spread of resistance genes [58]. A study conducted on swine feces and downstream water in China suggested that the effluent of animal farms contributes to the presence of ESBL-producing *E. coli* in river aquatic environments [59]. A recent study in Zhejiang, China also showed that these *mcr-1* plasmids in the river are closely associated with *E. coli* strains with pig and human origins [60].

IncX4 plasmids are one of the three major types of *mcr-1*-carrying plasmids, including IncX4, IncI2, and IncHI2, and account for over 90% of the reported *mcr-1* distributed worldwide [61]. It has been reported that IncX4 plasmids confer competitive fitness to host bacteria, are more transmissible at 30–42 °C [62], and can be stably maintained in host bacteria [41]. These features may be responsible for the significant role of IncX4 plasmids in *mcr-1* dissemination. The cessation of colistin use as a feed additive for animals in China has significantly decreased the prevalence of *mcr-1* in farmed pigs nationally, including IncX4 plasmid-carrying *mcr-1*, which may contribute to a concomitant decline of the distribution of *mcr-1* in human carriers [63]. These findings indicate that antibiotic selection pressure is a major driving force of *mcr-1* dissemination; thus, the withdrawal of colistin from animal feeds would be an effective strategy for controlling the dissemination of *mcr-1* in humans, animals, and the natural environment.

In our *mcr-1* positive *E. coli*, no known virulence factor was detected, excluding the *ompT* gene, and all were classified as phylogenetic group A, suggesting the low virulence potential of these bacteria. Although they have low potential to cause infections, these riverine strains could serve as an environmental reservoir of colistin resistance for future spread to pathogenic strains through conjugative horizontal transfer [64].

The insertion of the Tn3 family transposon in pEC1281 was first identified in the pEC1279-like IncX4 plasmids, suggesting that transposon insertion is a local gene transfer event that may have occurred recently; thus, the resulting plasmid has not yet been broadly spread. Whether the genes encoded in the transposon contribute to plasmid transmission and stability, and whether their presence confers advantages to bacterial survival in natural environments, are yet to be elucidated. Transposons and insertion sequences contribute to the mobilization of antibiotic resistance genes [65]. The new transposon insertion in the *mcr-1*-carrying plasmid may further potentiate the dissemination of colistin resistance.

The limitations of this study were that we only detected *mcr-1* in *E. coli* isolates in the river and we did not determine the *mcr-1* status of other pathogens. No selective primary isolation of colistin resistant *E. coli* was performed, and the real occurrence of *mcr-1* carrying *E. coli* could be much more prevalent. Other limitations included that the sampling sizes of rivers may differ and we did not have water level data for each sampling site. The water level of rivers varies significantly between the wet and dry seasons. We do not know if the water level is a factor for the spread of *mcr-1* positive *E. coli*. We did not conduct longitudinal surveillance of *mcr-1*-positive E. coli in the river. Some studies suggest that the surveillance of population-level antibiotic resistance prevalence could be informative as an early warning of human pathogens [66]. Real-time water quality monitoring systems and removed/relocated livestock may aid in reducing pollutants from agricultural areas [67].

## 5. Conclusions

The ecological analysis and plasmid sequences suggest the spread of *mcr-1* plasmids between livestock and the riverine environment. The *mcr-1*-positive *E. coli* isolates belonged to phylogroup A, with low virulence potential. The presence of colistin-resistant strains in rivers may lead to the spread of *mcr-1* among commensal *E. coli* strains in the aquatic environment and pose a further public health risk. There is an indispensable need for the survey of natural habitats or associated environments to better understand the dissimilation of colistin-resistance genes.

## Figures and Tables

**Table 1:** The antimicrobial drug susceptibility and STs in seven *mcr-1*-positive isolates.

NO	STX	TMZ	CIP	TET	MER	AZI	NAL	CTX	CHL	TIG	CAZe	COL	AMP	GM	ST
EC1278	>1024	≤0.25	≤0.015	>64	≤0.03	≤2	≤4	≤0.25	=32	≤0.25	≤0.5	=8	>64	≤0.5	3661
EC1279	>1024	>32	=0.25	>64	≤0.03	=4	>128	≤0.25	=128	=1	≤0.5	=8	>64	≤0.5	3661
EC1280	=16	=0.5	≤0.015	=64	≤0.03	=8	≤4	≤0.25	≤8	=0.5	≤0.5	=8	=2	≤0.5	3661
EC1281	>1024	>32	=0.25	>64	≤0.03	≤2	=128	≤0.25	=128	≤0.25	≤0.5	=8	>64	=1	877
EC1282	>1024	>32	=0.12	=64	≤0.03	=8	=8	≤0.25	=128	=0.5	≤0.5	≤1	>64	=1	155
EC1283	=16	=0.5	≤0.015	=4	≤0.03	=4	≤4	≤0.25	>128	≤0.25	≤0.5	=4	>64	=8	6732
EC1284	=16	=0.5	=0.03	≤2	≤0.03	=4	≤4	≤0.25	≤8	≤0.25	≤0.5	≤1	=4	≤0.5	7149

STX-TMZ; CIP: ciprofloxacin; TET: tetracycline; MER: meropenem; AZI: azithromycin; NAL: nalidixic acid; CTX: cefotaxime; CHL: chloroamphenicol; TIG: tigecycline; CAZ: ceftazidime; COL: colistin; AMP: ampicillin; GM: gentamicin.

**Table 2:** Independent *t*-test of the *mcr-1*-positive and *mcr-1*-negative *E. coli* sites.

Parameter	*mcr-1*-Positive *E. coli* Site	*mcr-1*-Negative *E. coli* Site	*p*-Value
	N = 7	N = 112	
Pollution index (mean ± SD)	4.08 ± 2.31	2.84 ± 1.71	0.073
Air temperature (mean ± SD)	20.59 ± 6.59	21.3 ± 4.78	0.712
Water temperature (mean ± SD)	21.02 ± 5.68	20.38 ± 3.65	0.661
PH (mean ± SD)	7.97 ± 0.36	7.81 ± 0.53	0.421
Coliform number (mean ± SD)	252,657 ± 375,707	169,949 ± 636,844	0.735
Chickens stocking density (birds/km^2^; mean ± SD)	5354 ± 4472	2956 ± 7007	0.223
Pigs stocking density (herds/km^2^; mean ± SD)	746 ± 1174	185 ± 421	0.254
Cows stocking density (herds/km^2^; mean ± SD)	45 ± 112	4.6 ± 17.4	0.372
Human population density (people/km^2^; mean ± SD)	1096 ± 1011	1680 ± 3533	0.665

**Table 3:** Chi-square test of the *mcr-1*-positive and *mcr-1*-negative *E. coli* sites.

Categorical Parameter	*mcr-1*-Positive *E. coli* Site (*n* = 7)	*mcr-1*-neGative *E. coli* Site (*n* = 112)	*p*-Value
Chicken stocking density (No./km^2^)			0.028 *
<1000	1 (14.3)	67 (59.8)	
1000–5000	3 (42.9)	31 (27.7)	
>5000	3 (42.9)	14 (12.5)	
	0	50	
Pig stocking density (No./km^2^)			0.012 *
<100	4 (57.1)	73 (65.2)	
100–1000	1 (14.3)	35 (31.2)	
>1000	2 (28.6)	4 (3.6)	
Cow stocking density (No./km^2^)			0.613
0–5	5 (71.4)	95 (84.8)	
6–10	1 (14.3)	7 (6.2)	
≥11	1 (14.3)	10 (8.9)	
River pollution severity			0.542
Unpolluted	1 (14.3)	42 (37.5)	
Negligibly polluted	2 (28.6)	23 (20.5)	
Moderately polluted	3 (42.9)	41 (36.6)	
Severely polluted	1 (14.3)	6 (5.4)	
Human population density			1.000
<=1000 people/km^2^	5 (71.4)	70 (62.5)	
>1000 people/km^2^	2 (28.6)	42 (37.5)	

* *p*-Value < 0.05.

**Table 4:** Examples of pEC1279-like plasmids.

Plasmid	Source	Strain	Regions	Size (bp)	Accession #	Reference
pKP15450-MCR-1	Human	*K. pneumoniae*	Taiwan	33,309	MH715959.1	
pNG14043	Human	*Salmonella*	Taiwan	33,308	KY120364.1	[36]
p2017.19.01CC	Human	*E. coli*	Vietnam	33,309	LC511660.1	[37]
p31349	Human	*E. coli*	Switzerland	33,303	KY689634.1	[38]
pmcr1_IncX4	Human	*K. pneumoniae*	China	33,287	KU761327.1	[39]
pMCR-1_Msc	Human	*E. coli*	Russia	33,310	MK172815.1	[40]
pWI2-mcr	Human	*E. coli*	France	33,304	LT838201.1	[41]
pICBEC12-3mcr	Avian	*E. coli*	Brazil	33,304	CP021419.1	
pMcp0271	Chicken meat	*E. coli*	Switzerland	33,303	KY565556	[38]
pHNSHP10	Swine	*E. coli*	China	33,309	MF774182.1	[42]
pNCYU-24-74-6_MCR1	Swine	*E. coli*	Taiwan	33,300	CP042644.1	[7]
pCSZ4	Pork	*E. coli*	China	33,309	KX711706.1	[43]
pMCR_WCHEC1618	Wastewater	*E. coli*	China	33,309	KY463454.1	[44]
pB2	Wastewater	*E. coli*	Japan	33,309	LC479085.1	[45]

# NCBI GenBank database accession numbers.

## Data Availability

Please contact author for data request.

## Supplementary Materials

The following are available online at https://www.mdpi.com/article/10.3390/microorganisms9040722/s1, Figure S1: *mcr-1*-positive site in the downstream area in Gaoping River, Table S1: Primers sequences used in this study.

## References

[1] Liu Y.-Y., Wang Y., Walsh T.R., Yi L.-X., Zhang R., Spencer J., Doi Y., Tian G., Dong B., Huang X. (2016). Emergence of plasmid-mediated colistin resistance mechanism MCR-1 in animals and human beings in China: A microbiological and molecular biological study. Lancet Infect. Dis..

[2] Nang S.C., Li J., Velkov T. (2019). The rise and spread of mcr plasmid-mediated polymyxin resistance. Crit. Rev. Microbiol..

[3] Wang R., Van Dorp L., Shaw L.P., Bradley P., Wang Q., Wang X., Jin L., Zhang Q., Liu Y., Rieux A. (2018). The global distribution and spread of the mobilized colistin resistance gene *mcr-1*. Nat. Commun..

[4] Wang Y., Tian G.-B., Zhang R., Shen Y., Tyrrell J.M., Huang X., Zhou H., Lei L., Li H.-Y., Doi Y. (2017). Prevalence, risk factors, outcomes, and molecular epidemiology of *mcr-1* -positive Enterobacteriaceae in patients and healthy adults from China: An epidemiological and clinical study. Lancet Infect. Dis..

[5] Schwarz S., Johnson A.P. (2016). Transferable resistance to colistin: A new but old threat: Table 1. J. Antimicrob. Chemother..

[6] Zhou H.-W., Zhang T., Ma J.-H., Fang Y., Wang H.-Y., Huang Z.-X., Wang Y., Wu C., Chen G.-X. (2017). Occurrence of Plasmid- and Chromosome-Carried *mcr-1* in Waterborne Enterobacteriaceae in China. Antimicrob. Agents Chemother..

[7] Liu J.-Y., Liao T.-L., Huang W.-C., Liu Y.-M., Wu K.-M., Lauderdale T.-L., Tsai S.-F., Kuo S.-C., Kuo H.-C. (2020). Increased *mcr-1* in pathogenic *Escherichia coli* from diseased swine, Taiwan. J. Microbiol. Immunol. Infect..

[8] Wu P.-C., Wang J.-L., Hsueh P.-R., Lin P.-H., Cheng M.-F., Huang I.-F., Chen Y.-S., Lee S.S.-J., Mar G.-Y., Yu H.-C. (2019). Prevalence and risk factors for colonization by extended-spectrum β-lactamase-producing or ST 131 *Escherichia coli* among asymptomatic adults in community settings in Southern Taiwan. Infect. Drug Resist..

[9] Jean S.-S., Lu M.-C., Shi Z.-Y., Tseng S.-H., Wu T.-S., Lu P.-L., Shao P.-L., Ko W.-C., Wang F.-D., Hsueh P.-R. (2018). In vitro activity of ceftazidime–avibactam, ceftolozane–tazobactam, and other comparable agents against clinically important Gram-negative bacilli: Results from the 2017 Surveillance of Multicenter Antimicrobial Resistance in Taiwan (SMART). Infect. Drug Resist..

[10] Kuo S.-C., Huang W.-C., Wang H.-Y., Shiau Y.-R., Cheng M.-F., Lauderdale T.-L. (2016). Colistin resistance gene *mcr-1* in *Escherichia coli* isolates from humans and retail meats, Taiwan. J. Antimicrob. Chemother..

[11] Chen K., Chan E.W.-C., Xie M., Ye L., Dong N., Chen S. (2017). Widespread distribution of *mcr-1*-bearing bacteria in the ecosystem, 2015 to 2016. Euro Surveill..

[12] Wang R.-N., Zhang Y., Cao Z.-H., Wang X.-Y., Ma B., Wu W.-B., Hu N., Huo Z.-Y., Yuan Q.-B. (2019). Occurrence of super antibiotic resistance genes in the downstream of the Yangtze River in China: Prevalence and antibiotic resistance profiles. Sci. Total. Environ..

[13] Anyanwu M.U., Jaja I.F., Nwobi O.C. (2020). Occurrence and Characteristics of Mobile Colistin Resistance (mcr) Gene-Containing Isolates from the Environment: A Review. Int. J. Environ. Res. Public Health.

[14] Tuo H., Yang Y., Tao X., Liu D., Li Y., Xie X., Li P., Gu J., Kong L., Xiang R. (2018). The Prevalence of Colistin Resistant Strains and Antibiotic Resistance Gene Profiles in Funan River, China. Front. Microbiol..

[15] Yang D., Qiu Z., Shen Z., Zhao H., Jin M., Li H., Liu W., Li J.-W. (2017). The Occurrence of the Colistin Resistance Gene *mcr-1* in the Haihe River (China). Int. J. Environ. Res. Public Health.

[16] Yu C.Y., Ang G.Y., Chin P.S., Ngeow Y.F., Yin W.-F., Chan K.G. (2016). Emergence of *mcr-1*-mediated colistin resistance in *Escherichia coli* in Malaysia. Int. J. Antimicrob. Agents.

[17] Caltagirone M., Nucleo E., Spalla M., Zara F., Novazzi F., Marchetti V.M., Piazza A., Bitar I., De Cicco M., Paolucci S. (2017). Occurrence of Extended Spectrum β-Lactamases, KPC-Type, and MCR-1.2-Producing Enterobacteriaceae from Wells, River Water, and Wastewater Treatment Plants in Oltrepò Pavese Area, Northern Italy. Front. Microbiol..

[18] Zurfluh K., Poirel L., Nordmann P., Nüesch-Inderbinen M., Hächler H., Stephan R. (2016). Occurrence of the Plasmid-Borne *mcr-1* Colistin Resistance Gene in Extended-Spectrum-β-Lactamase-Producing Enterobacteriaceae in River Water and Imported Vegetable Samples in Switzerland. Antimicrob. Agents Chemother..

[19] Sun P.L., Hawkins W.E., Overstreet R.M., Brown-Peterson N.J. (2009). Morphological Deformities as Biomarkers in Fish from Contaminated Rivers in Taiwan. Int. J. Environ. Res. Public Health.

[20] Chen Y.-C., Yeh H.-C., Wei C. (2012). Estimation of River Pollution Index in a Tidal Stream Using Kriging Analysis. Int. J. Environ. Res. Public Health.

[21] Putri M.S.A., Lou C.-H., Syai’In M., Ou S.-H., Wang Y.-C. (2018). Long-Term River Water Quality Trends and Pollution Source Apportionment in Taiwan. Water.

[22] Chen P.-A., Hung C.-H., Huang P.-C., Chen J.-R., Huang I.-F., Chen W.-L., Chiou Y.-H., Hung W.-Y., Wang J.-L., Cheng M.-F. (2016). Characteristics of CTX-M Extended-Spectrum β-Lactamase-Producing *Escherichia coli* Strains Isolated from Multiple Rivers in Southern Taiwan. Appl. Environ. Microbiol..

[23] Hsu P.-C., Chen C.-S., Wang S., Hashimoto M., Huang W.-C., Teng C.-H. (2020). Identification of MltG as a Prc Protease Substrate Whose Dysregulation Contributes to the Conditional Growth Defect of Prc-Deficient *Escherichia coli*. Front. Microbiol..

[24] Kado I.C., Liu S.T. (1981). Rapid procedure for detection and isolation of large and small plasmids. J. Bacteriol..

[25] Sambrook J., Russell D.W. (2001). Molecular Cloning: A Laboratory Manual.

[26] Tanizawa Y., Fujisawa T., Kaminuma E., Nakamura Y., Arita M. (2016). DFAST and DAGA: Web-based integrated genome annotation tools and resources. Biosci. Microbiota Food Health.

[27] Siguier P. (2006). ISfinder: The reference centre for bacterial insertion sequences. Nucleic Acids Res..

[28] Clermont O., Bonacorsi S., Bingen E. (2000). Rapid and Simple Determination of the *Escherichia coli* Phylogenetic Group. Appl. Environ. Microbiol..

[29] Chapman T.A., Wu X.-Y., Barchia I., Bettelheim K.A., Driesen S., Trott D., Wilson M., Chin J.J.-C. (2006). Comparison of Virulence Gene Profiles of *Escherichia coli* Strains Isolated from Healthy and Diarrheic Swine. Appl. Environ. Microbiol..

[30] Johnson J.R., Stell A.L. (2000). Extended Virulence Genotypes of *Escherichia coli* Strains from Patients with Urosepsis in Relation to Phylogeny and Host Compromise. J. Infect. Dis..

[31] Johnson T.J., Wannemuehler Y., Doetkott C., Rosenberger S.C., Nolan L.K. (2008). Identification of Minimal Predictors of Avian Pathogenic *Escherichia coli* Virulence for Use as a Rapid Diagnostic Tool. J. Clin. Microbiol..

[32] Mao B.-H., Chang Y.-F., Scaria J., Chang C.-C., Chou L.-W., Tien N., Wu J.-J., Tseng C.-C., Wang M.-C., Hsu Y.-M. (2011). Identification of *Escherichia coli* Genes Associated with Urinary Tract Infections. J. Clin. Microbiol..

[33] Huang W.-C., Liao Y.-J., Hashimoto M., Chen K.-F., Chu C., Hsu P.-C., Wang S., Teng C.-H. (2020). cjrABC-senB hinders survival of extraintestinal pathogenic *E. coli* in the bloodstream through triggering complement-mediated killing. J. Biomed. Sci..

[34] Huang W.-C., Lin C.-Y., Hashimoto M., Wu J.-J., Wang M.-C., Lin W.-H., Chen C.-S., Teng C.-H. (2020). The role of the bacterial protease Prc in the uropathogenesis of extraintestinal pathogenic *Escherichia coli*. J. Biomed. Sci..

[35] Chu C., Chiu C.-H., Wu W.-Y., Chu C.-H., Liu T.-P., Ou J.T. (2001). Large Drug Resistance Virulence Plasmids of Clinical Isolates of Salmonella enterica Serovar Choleraesuis. Antimicrob. Agents Chemother..

[36] Chiou C.-S., Chen Y.-T., Wang Y.-W., Liu Y.-Y., Kuo H.-C., Tu Y.-H., Lin A.-C., Liao Y.-S., Hong Y.-P. (2017). Dissemination of *mcr-1*-Carrying Plasmids among Colistin-Resistant Salmonella Strains from Humans and Food-Producing Animals in Taiwan. Antimicrob. Agents Chemother..

[37] Yamaguchi T., Kawahara R., Hamamoto K., Hirai I., Khong D.T., Nguyen T.N., Tran H.T., Motooka D., Nakamura S., Yamamoto Y. (2020). High Prevalence of Colistin-Resistant *Escherichia coli* with Chromosomally Carried *mcr-1* in Healthy Residents in Vietnam. mSphere.

[38] Donà V., Bernasconi O.J., Pires J., Collaud A., Overesch G., Ramette A., Perreten V., Endimiani A. (2017). Heterogeneous Genetic Location of *mcr-1* in Colistin-Resistant *Escherichia coli* Isolates from Humans and Retail Chicken Meat in Switzerland: Emergence of *mcr-1*-Carrying IncK2 Plasmids. Antimicrob. Agents Chemother..

[39] Liang C., Yang Y., Miao M., Chavda K.D., Mediavilla J.R., Xie X., Feng P., Tang Y.-W., Kreiswirth B.N., Chen L. (2016). Complete Sequences of *mcr-1*-Harboring Plasmids from Extended-Spectrum-β-Lactamase- and Carbapenemase-Producing Enterobacteriaceae. Antimicrob. Agents Chemother..

[40] Ageevets V., Lazareva I., Mrugova T., Gostev V., Lobzin Y., Sidorenko S. (2019). IncX4 plasmids harbouring *mcr-1* genes: Further dissemination. J. Glob. Antimicrob. Resist..

[41] Beyrouthy R., Robin F., Lessene A., Lacombat I., Dortet L., Naas T., Ponties V., Bonnet R. (2017). MCR-1 and OXA-48 In Vivo Acquisition in KPC-Producing *Escherichia coli* after Colistin Treatment. Antimicrob. Agents Chemother..

[42] Wu R., Yi L.-X., Yu L.-F., Wang J., Liu Y., Chen X., Lv L., Yang J., Liu J.-H. (2018). Fitness Advantage of *mcr-1*–Bearing IncI2 and IncX4 Plasmids in Vitro. Front. Microbiol..

[43] Sun J., Fang L.-X., Wu Z., Deng H., Yang R.-S., Li X.-P., Li S.-M., Liao X.-P., Feng Y., Liu Y.-H. (2017). Genetic Analysis of the IncX4 Plasmids: Implications for a Unique Pattern in the *mcr-1* Acquisition. Sci. Rep..

[44] Zhao F., Feng Y., Lü X., McNally A., Zong Z. (2017). Remarkable Diversity of *Escherichia coli* Carrying *mcr-1* from Hospital Sewage with the Identification of Two New *mcr-1* Variants. Front. Microbiol..

[45] Hayashi W., Tanaka H., Taniguchi Y., Iimura M., Soga E., Kubo R., Matsuo N., Kawamura K., Arakawa Y., Nagano Y. (2019). Acquisition of *mcr-1* and Cocarriage of Virulence Genes in Avian Pathogenic *Escherichia coli* Isolates from Municipal Wastewater Influents in Japan. Appl. Environ. Microbiol..

[46] Creighton J., Anderson T., Howard J., Dyet K., Ren X., Freeman J. (2019). Co-occurrence of *mcr-1* and mcr-3 genes in a single *Escherichia coli* in New Zealand. J. Antimicrob. Chemother..

[47] Zurfluh K., Klumpp J., Nüesch-Inderbinen M., Stephan R. (2016). Full-Length Nucleotide Sequences of *mcr-1*-Harboring Plasmids Isolated from Extended-Spectrum-β-Lactamase-Producing *Escherichia coli* Isolates of Different Origins. Antimicrob. Agents Chemother..

[48] Yang L., Lin Y., Lu L., Xue M., Ma H., Guo X., Wang K., Li P., Du X., Qi K. (2020). Coexistence of Two blaNDM–5 Genes Carried on IncX3 and IncFII Plasmids in an *Escherichia coli* Isolate Revealed by Illumina and Nanopore Sequencing. Front. Microbiol..

[49] Akiba M., Sekizuka T., Yamashita A., Kuroda M., Fujii Y., Murata M., Lee K.-I., Joshua D.I., Balakrishna K., Bairy I. (2016). Distribution and Relationships of Antimicrobial Resistance Determinants among Extended-Spectrum-Cephalosporin-Resistant or Carbapenem-Resistant *Escherichia coli* Isolates from Rivers and Sewage Treatment Plants in India. Antimicrob. Agents Chemother..

[50] Podschun R., Ullmann U. (1998). Klebsiella spp. as Nosocomial Pathogens: Epidemiology, Taxonomy, Typing Methods, and Pathogenicity Factors. Clin. Microbiol. Rev..

[51] Picard B., Garcia J.S., Gouriou S., Duriez P., Brahimi N., Bingen E., Elion J., Denamur E. (1999). The Link between Phylogeny and Virulence in *Escherichia coli* Extraintestinal Infection. Infect. Immun..

[52] Checcucci A., Trevisi P., Luise D., Modesto M., Blasioli S., Braschi I., Mattarelli P. (2020). Exploring the Animal Waste Resistome: The Spread of Antimicrobial Resistance Genes Through the Use of Livestock Manure. Front. Microbiol..

[53] He Y., Yuan Q., Mathieu J., Stadler L., Senehi N., Sun R., Alvarez P.J.J. (2020). Antibiotic resistance genes from livestock waste: Occurrence, dissemination, and treatment. NPJ Clean Water.

[54] Egao L., Ehu J., Zhang X., Ewei L., Eli S., Emiao Z., Echai T. (2015). Application of swine manure on agricultural fields contributes to extended-spectrum β-lactamase-producing *Escherichia coli* spread in Tai’an, China. Front. Microbiol..

[55] Curriero F.C., Patz J.A., Rose J.B., Lele S. (2001). The Association Between Extreme Precipitation and Waterborne Disease Outbreaks in the United States, 1948–1994. Am. J. Public Health.

[56] Rousham E.K., Unicomb L., Islam M.A. (2018). Human, animal and environmental contributors to antibiotic resistance in low-resource settings: Integrating behavioural, epidemiological and One Health approaches. Proc. R. Soc. B Boil. Sci..

[57] Trung N.V., Carrique-Mas J.J., Hoang N.N., Mai H.H., Tuyen H.T., Campbell J.I., Nhung N.T., Nhung H.N., Van Minh P., Wagenaar J.A. (2015). Prevalence and risk factors for carriage of antimicrobial-resistant *Escherichia coli* on household and small-scale chicken farms in the Mekong Delta of Vietnam. J. Antimicrob. Chemother..

[58] Gao L., Hu J., Zhang X., Ma R., Gao J., Li S., Zhao M., Miao Z., Chai T. (2014). Dissemination of ESBL-Producing *Escherichia coli* of Chicken Origin to the Nearby River Water. J. Mol. Microbiol. Biotechnol..

[59] Li S., Song W., Zhou Y., Tang Y., Gao Y., Miao Z. (2015). Spread of extended-spectrum beta-lactamase-producing *Escherichia coli* from a swine farm to the receiving river. Environ. Sci. Pollut. Res..

[60] Zhu L., Zhou Z., Liu Y., Lin Z., Shuai X., Xu L., Chen H. (2020). Comprehensive Understanding of the Plasmid-Mediated Colistin Resistance Gene *mcr-1* in Aquatic Environments. Environ. Sci. Technol..

[61] Matamoros S., Van Hattem J.M., Arcilla M.S., Willemse N., Melles D.C., Penders J., Vinh T.N., Hoa N.T., Bootsma M.C.J., Van Genderen P.J. (2017). Global phylogenetic analysis of *Escherichia coli* and plasmids carrying the *mcr-1* gene indicates bacterial diversity but plasmid restriction. Sci. Rep..

[62] Lo W.-U., Chow K.-H., Law P.Y., Ng K.-Y., Cheung Y.-Y., Lai E.L., Ho P.-L. (2014). Highly conjugative IncX4 plasmids carrying bla CTX-M in *Escherichia coli* from humans and food animals. J. Med. Microbiol..

[63] Shen C., Zhong L.-L., Yang Y., Doi Y., Paterson D.L., Stoesser N., Ma F., Ahmed M.A.E.-G.E.-S., Feng S., Huang S. (2020). Dynamics of *mcr-1* prevalence and *mcr-1*-positive *Escherichia coli* after the cessation of colistin use as a feed additive for animals in China: A prospective cross-sectional and whole genome sequencing-based molecular epidemiological study. Lancet Microbe.

[64] Johura F.-T., Tasnim J., Barman I., Biswas S.R., Jubyda F.T., Sultana M., George C.M., Camilli A., Seed K.D., Ahmed N. (2020). Colistin-resistant *Escherichia coli* carrying *mcr-1* in food, water, hand rinse, and healthy human gut in Bangladesh. Gut Pathog..

[65] Partridge S.R., Kwong S.M., Firth N., Jensen S.O. (2018). Mobile Genetic Elements Associated with Antimicrobial Resistance. Clin. Microbiol. Rev..

[66] Huijbers P.M., Flach C.-F., Larsson D.J. (2019). A conceptual framework for the environmental surveillance of antibiotics and antibiotic resistance. Environ. Int..

[67] Kao C., Wu F., Chen K., Lin T., Yen Y., Chiang P. (2003). Pollutant sources investigation and remedial strategies development for the Kaoping River Basin, Taiwan. Water Sci. Technol..

