# Supplementary material for: A Large Spatial Survey of Colistin-Resistant Gene mcr-1-Carrying E. coli in Rivers across Taiwan
Source: Microorganisms. 2021 Mar 31;9(4):722. doi: 10.3390/microorganisms9040722 (PMC8066897; doi:10.3390/microorganisms9040722)

Table S1. Primers sequences used in this study.

Gene	Sequence（5' to 3'）	Amplicon size（bp）	Reference
hlyF	GGCCACAGTCGTTTAGGGTGCTTACC<br>GGCGGTTTAGGCATTCCGATACTCAG	450	(Johnson et al., 2008)
iutA	GGCTGGACATCATGGGAACTGG<br>GTCGGGGAACGGGTAGAATCG	302	
iss	CAGCAACCCGAACCACTTGATG<br>AGCATTGCCAGAGCGGCAGAA	323	
ibeA	GACTGGCACGCGAAATAGAA<br>AGCTGGACTCGCCCAATAAT	793	(Mao et al., 2012)
usp	ATGGTCGCTCAGTGGCATAC<br>CAGCGAGTTCCTGGTGAAAG	636	
sat	ACGGTCAGGGATTTACATTT<br>GCTATTTGGCTGTTATGTGC	1099	
ompT	ATCTAGCCGAAGAAGGAGGC<br>CCCGGGTCATAGTGTTCATC	559	(Chapman et al., 2006)
chuA	GACGAACCAACGGTCAGGAT<br>TGCCGCCAGTACCAAAGACA	279	
iha	CTGGCGGAGGCTCTGAGATCA<br>TCCTTAAGCTCCCGCGGCTGA	827	
ireA	GATGACTCAGCCACGGGTAA<br>CCAGGACTCACCTCACGAAT	254	
hlyA	AACAAGGATAAGCACTGTTCTGGCT<br>ACCATATAAGCGGTCATTCCCGTCA	1177	
iroN	AAGTCAAAGCAGGGGTTGCCCG<br>GACGCCGACATTAAGACGCAG	665	
sfaS	GTGGATACGACGATTACTGTG<br>CCGCCAGCATTCCCTGTATTC	240	
afa/draBC	GGCAGAGGGCCGGCAACAGGC<br>CCCGTAACGCGCCAGCATCTC	559	
cnf1	AAGATGGAGTTTCCTATGCAGGAG<br>CATTCAGAGTCCTGCCCTCATTATT	498	

Reference:

Chapman, T. A., Wu, X. Y., Barchia, I., Bettelheim, K. A., Driesen, S., Trott, D., . . . Chin, J. J. (2006). Comparison of virulence gene profiles of Escherichia coli strains isolated from healthy and diarrheic swine. *Appl Environ Microbiol, 72*(7), 4782-4795. doi:10.1128/AEM.02885-05

Johnson, T. J., Wannemuehler, Y., Doetkott, C., Johnson, S. J., Rosenberger, S. C., & Nolan, L. K. (2008). Identification of minimal predictors of avian pathogenic Escherichia coli virulence for use as a rapid diagnostic tool. *J Clin Microbiol, 46*(12), 3987-3996. doi:10.1128/JCM.00816-08

Mao, B. H., Chang, Y. F., Scaria, J., Chang, C. C., Chou, L. W., Tien, N., . . . Teng, C. H. (2012). Identification of Escherichia coli genes associated with urinary tract infections. *J Clin Microbiol, 50*(2), 449-456. doi:10.1128/JCM.00640-11

Figure S1: mcr-1-positive site in the downstream area in Gaoping River

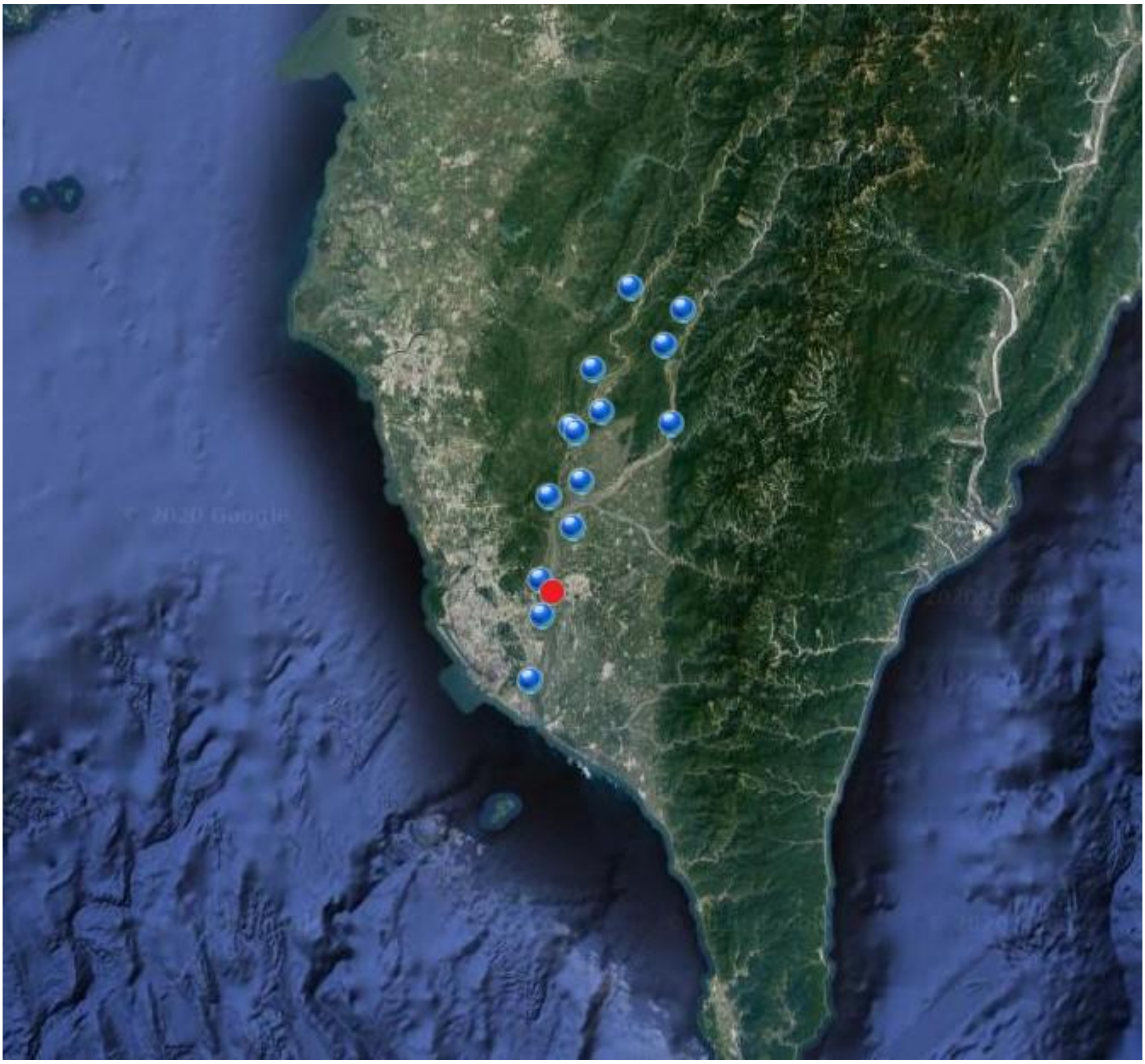

Supplement: Supplementary file 1 [file microorganisms-09-00722-s001.pdf]
